# Supplementary material for: Neuropilin-1 as a Key Molecule for Renal Recovery in Lupus Nephritis: Insights from an NZB/W F1 Mouse Model
Source: Int J Mol Sci. 2024 Oct 22;25(21):11364. doi: 10.3390/ijms252111364 (PMC11545582; doi:10.3390/ijms252111364)

Supporting Information

Neuropilin-1 as a Key Molecule for Renal Recovery in Lupus Nephritis: Insights from NZB/W F1 Mouse Model

Sebastian Sandoval [1], Cristina Solé [1,*], Blanca Joseph-Mullol [1], Maria Royo [1], Teresa Moliné [2], Alejandra Gabaldón [2] and Josefina Cortés-Hernández [1]

1 Rheumatology Research Group—Lupus Unit, Vall d'Hebron University Hospital, Vall d'Hebron Research Institute (VHIR), Universitat Autònoma de Barcelona (UAB), 08193 Barcelona, Spain; 2 Department of Pathology, Vall d'Hebron University Hospital, 08035 Barcelona, Spain; *Correspondence: cristina.sole@vhir.org ; Tel: +34-934894045

INDEX

1. SI Material and Methods 3
2. Supplementary figures 5

1. SI Material and Methods

Kidney Histology and Immunofluorescence

Kidneys were harvested from mice and fixed in 10% formalin. After fixation, tissues were dehydrated using graded alcohol concentrations of 70%, 80%, 95%, and 99%, and subsequently embedded in paraffin. Sections were cut at 5µm thickness using a microtome for either hematoxylin and eosin (H&E) or immunofluorescence staining. For H&E staining, hematoxylin (ThermoFisher, Carlsbad, CA, USA) was applied for 10 minutes, followed by two quick dips in 0.3% acid alcohol. The tissues were then counterstained with eosin Y (ThermoFisher, Carlsbad, CA, USA) for 2 minutes, washed in running water, dehydrated, and mounted. Microscopic images of the H&E-stained sections were acquired using an Olympus bright-field microscope equipped with a DP27 digital camera at 20× magnification.

For immunofluorescence, paraffin-embedded kidney sections (5µm) were deparaffinized in xylene for 10 minutes and rehydrated through a graded ethanol series (99%, 95%, 80%, and 70%) for 2–3 minutes each. The sections were fixed in 4% paraformaldehyde (Thermo Scientific, Waltham, MA, USA) before staining. Antigen retrieval was performed using proteinase K (Thermo Scientific, Waltham, MA, USA) for 20 minutes at room temperature. To block non-specific binding, the sections were incubated with 5% bovine serum albumin (BSA) for 1 hour at room temperature.

Primary antibodies were applied overnight at 4°C at the following dilutions: rabbit anti-mouse IgG (1:250, GTX26709, GeneTex, Irvine, CA, USA), C3 (1:250, GTX101316, GeneTex, Irvine, CA, USA), and NRP-1 (1:50, sc-5307, Santa Cruz Biotechnology, Dallas, TX, USA). After incubation, the sections were washed with phosphate-buffered saline (PBS) containing 0.25% Triton X-100. A secondary goat anti-rabbit antibody conjugated with DyLight488 (1:250, GTX213110-04, GeneTex, Irvine, CA, USA) was applied for 2 hours at room temperature, followed by washing with PBS containing 0.25% Triton X-100 to remove unbound secondary antibodies. Slides were mounted using VECTASHIELD antifade mounting medium with DAPI (Vector Laboratories, Newark, CA, USA) and imaged with an Olympus BX61 fluorescence microscope. Fluorescence intensity was quantified using ImageJ Fiji software (version 1.45).

Evaluation of Immunofluorescence in Kidney Sections

Immunofluorescence staining in kidney sections was evaluated by two blinded pathology experts from Vall d'Hebron University Hospital in collaboration with the research team from the SLE Unit, using an Olympus BX61 fluorescence microscope. Five images were captured from each tissue sample (n = 5) and analyzed to assess staining intensity using ImageJ Fiji software (version 1.45). Regions of interest (ROIs) were selected for fluorescence intensity quantification. For IgG and C3 staining, all glomeruli were included as ROIs to assess deposition within the glomerular region. For NRP-1 staining, five ROIs were selected, covering both glomerular and tubular sections.

The mean gray value was used to represent the average fluorescence intensity within the selected ROIs. To account for non-specific signal, background fluorescence was subtracted by selecting an area without any detectable fluorescence in each image. Fluorescence intensity was then normalized using negative control staining. The fold change in intensity was calculated by dividing each sample's mean gray value by the mean gray value of the negative control.

2. Supplementary Figures

Supplementary Figure S1. Histological scoring of kidney tissue of NZB/W F1 mice. Indices of activity (**A**) and chronicity (**B**) in the renal tissue of mice from both CYC-treated and untreated groups were evaluated by pathologists at Vall d'Hebron Hospital using specific scales designed for lupus nephritis in murine models. No significant differences were observed (p > 0.05).

A

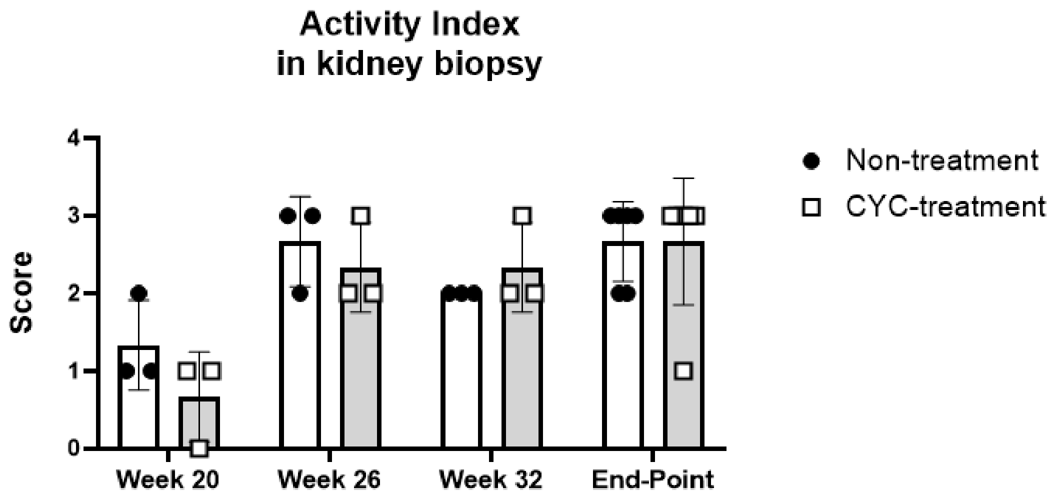

B

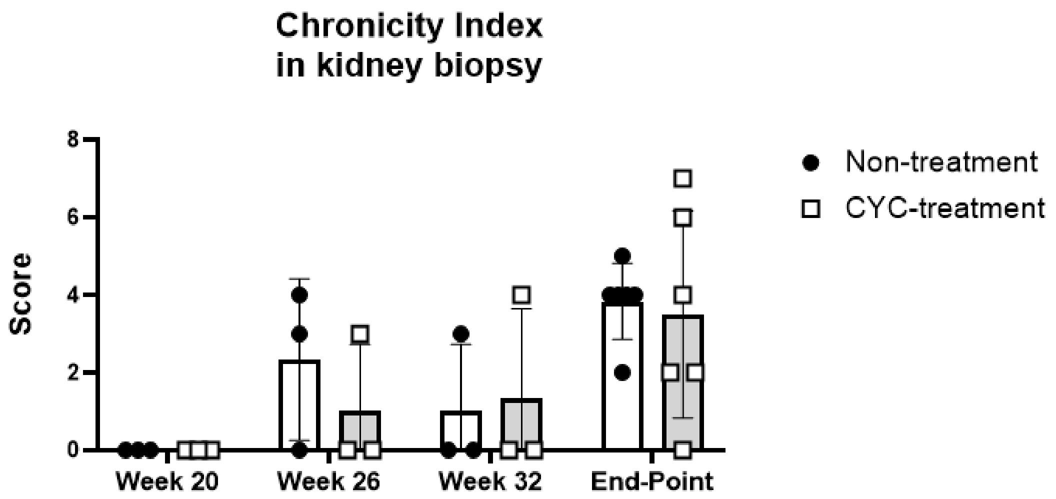

Supplementary Figure S2. Urinary NRP-1 (uNRP-1) protein levels in NZB/W F1 mice during the study. Urinary NRP-1 levels were measured at Weeks 20, 26, 32, and at the study endpoint in both CYC-treated and untreated NZB/W F1 mouse groups. A Student's t-test was used to compare uNRP-1 levels between the treatment groups at each time point. *p < 0.05.

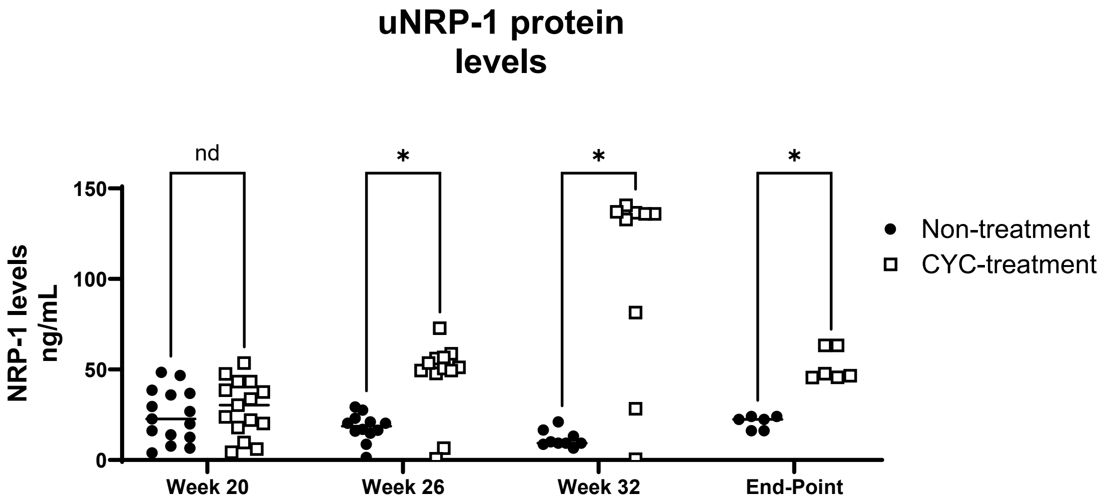

Supplementary Figure S3. **Study workflow.** NZB/W F1 mice were divided into two treatment groups: untreated and cyclophosphamide-treated (CYC). Kidney, blood, and urine samples were collected for analysis, as illustrated in the scheme.

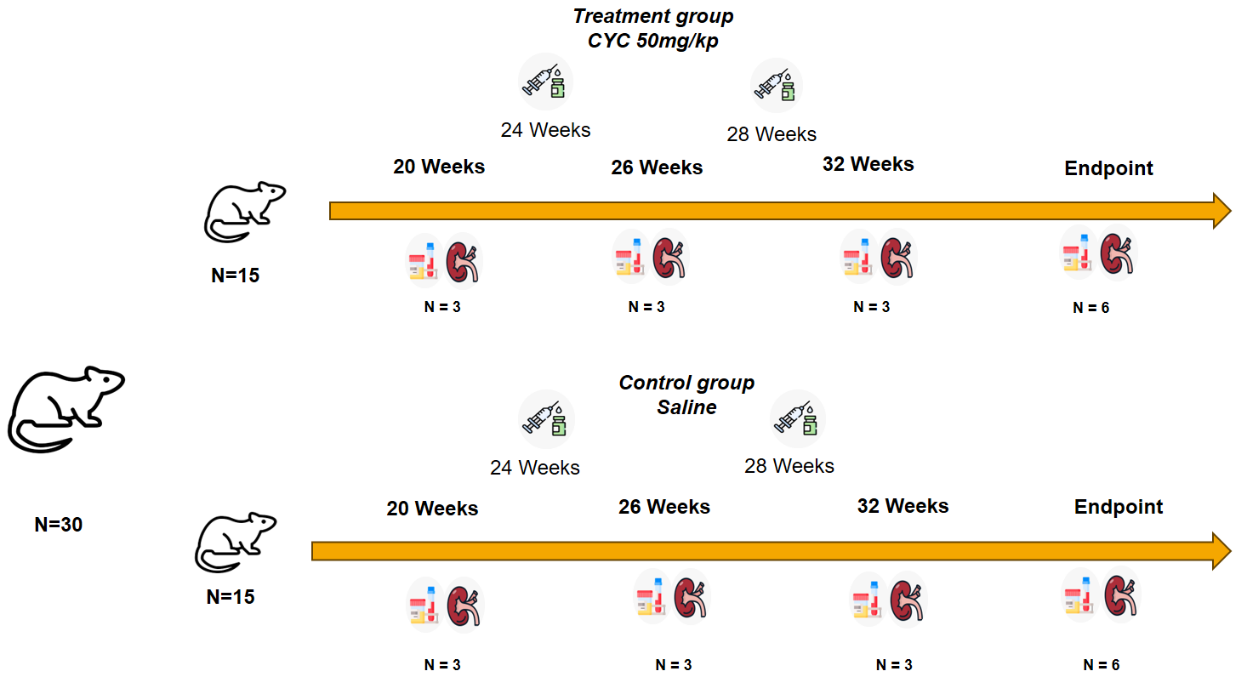

Supplement: Supplementary file 1 [file ijms-25-11364-s001.zip › ijms-3238339-supplementary.pdf]
